# Evaluation of potential drug-drug interactions and polypharmacy in hospitalized COVID-19 patients

**DOI:** 10.4314/ahs.v22i4.65

**Published:** 2022-12

**Authors:** Türkan Paşalı Kilit, Filiz Özyiğit, Sertaş Erarslan, Kevser Onbaşı

**Affiliations:** 1 Department of Internal Medicine, Kütahya Health Sciences University Faculty of Medicine, Kütahya, Turkey; 2 Kütahya, Turkey; 3 Department of Endocrinology, Kütahya Health Sciences University Faculty of Medicine, Kütahya, Turkey

**Keywords:** COVID-19, drug interactions, polypharmacy

## Abstract

**Background:**

Drugs that are used in COVID-19 infection, may interact with each other, as well as with the drugs for comorbidities, used concomitantly with COVID-19 treatment.

**Objectives:**

It is quite important to calculate and present the patients' exposure to clinically important potential drug-drug interactions (pDDIs). We aimed to investigate the pDDIs and the burden of polypharmacy in COVID-19.

**Methods:**

The medical records of 126 consecutive inpatients with COVID-19 treatment were retrospectively analyzed. The Lexi-interact database was used to investigate pDDIs.

**Results:**

According to the Lexi-interact database, 605 pDDIs were detected. Of these pDDIs, 23 (3.8%) were A risk category interaction, 186 (30.7%) were B risk category interaction, 339 (56%) were C risk category interaction, 54 (8.9%) were D risk category interaction, and 3 (0.5%) were X risk category interaction. Sixty-five-point five percent of pDDIs (n=396) were clinically important pDDIs (C, D, and X categories), and 69 patients (54.8%) had at least one clinically important pDDIs. The most interacting drug was hydroxychloroquine (n=171, 28.3%). Hydroxychloroquine was also the most interacting drug in the C risk category (n=101, 29.8%) and had 19 pDDIs with metformin, 16 pDDIs with beta-blockers, 13 pDDIs with acetylsalicylic acid, and 10 pDDIs with insulin in the C risk category. Enoxaparin was the most interacting drug (n=25, 46.3%) in the D risk category and most of them were with acetylsalicylic acid (n=12). The most common possible clinical manifestations of pDDIs were QT prolongation, hypoglycemia, and hemorrhage. One hundred and eighteen patients (93.6%) used five or more drugs daily. There was a significant positive correlation between the number of drugs prescribed to patients and the number of clinically important pDDIs (r=0.80, p<0.001).

**Conclusions:**

Clinically important pDDIs are common among COVID-19 patients and the majority of pDDIs require monitoring of therapy. COVID-19 patients should be closely observed for QT prolongation, hypoglycemia, and hemorrhage due to pDDIs during treatment.

## Introduction

In December 2019, a novel coronavirus was observed in the Wuhan city of China that could be presented with pneumonia. After the identification and characterization of this new coronavirus (severe acute respiratory syndrome coronavirus 2, SARS-CoV2), it resulted in a pandemic 1, 2. This SARS-CoV2 can enter host cells via a cell surface receptor called angiotensin-converting enzyme 2 (ACE-2), which is expressed in various human organs. In addition, to be a cause of pneumonia, SARS-CoV2 can also damage other systems and organs, including the heart, liver, and kidneys 3. Although not all pathophysiological mechanisms were understood, we know so far that patients with comorbidity (hypertension, diabetes mellitus, cardiovascular disease, chronic obstructive pulmonary disease, and chronic renal failure) and older age develop the more severe and mortal disease 4. Elderly patients are prone to coronavirus disease (COVID-19) and older people had the highest fatality rate since the beginning of the pandemic 5, 6. Also, polypharmacy is increased in the elderly due to multiple morbidities. For these reasons, it is very important to manage elderly COVID-19 patients properly, especially those with chronic accompanying diseases and who use multiple medications.

Chloroquine, hydroxychloroquine (HCQ), lopinavir/ritonavir, remdesivir, and favipiravir were used in the treatment of COVID-19 7–10. The use of chloroquine and HCQ have been ceased in this infection due to insufficient efficacy and potential risks. HCQ reduced neither mortality among hospitalized COVID-19 patients, nor the need or duration of mechanical ventilation. Taking HCQ to treat COVID-19 may increase the risk of heart rhythm problems, blood and lymph disorders, kidney injury, and liver failure. Consequently, they have been removed from the treatment algorithms. However, in some countries, their use was advised but restricted for suspected and symptomatic patients in the early phases of the disease 9.

The burden of the use of plenty of drugs and therefore potential drug-drug interactions (pDDIs) is a problem in COVID-19 patients. The prevalence of pDDIs was found to be high in studies conducted with patients with COVID-19 11. pDDIs occur between medications used in the course of COVID-19 infection and medications prescribed for the management of underlying comorbidities. Comorbidities and polypharmacy were found as risk factors independently associated with pDDIs development. Among hospitalized COVID-19 patients, HCQ and lopinavir/ritonavir have been associated with potentially severe pDDIs 12. pDDIs increase the risk of serious adverse drug reactions such as QT-prolongation, retinopathy, increased risk of infection, and hepatotoxicity 13. These adverse drug reactions may contribute to an increase in the risk of hospitalization, prolonged time to recovery, or death in extreme cases 14.

Clinically relevant pDDIs may be described as interference by using two drugs at the same time, irrelevant of developing an adverse event 15. Several electronic databases may be used as a tool for the evaluation of potentially harmful pDDIs. In interactions that fall into risk category C when classified according to one of these databases, the Lexi-Interact database, it is recommended that the treatment be closely monitored, even if the benefit derived from the combination of the two interacting drugs is generally greater than the risk from the interaction. According to the literature, C risk category pDDIs are the most frequently observed interactions 16. In this study, we aimed to assess the prevalence of pDDIs and determine the interacting drugs in hospitalized COVID-19 patients.

## Materials And Methods

### Study Population

One hundred and twenty-six patients between the ages of 26 and 87 who were hospitalized with the diagnosis of COVID-19 between April 2020 and June 2020, were included in our single-center, retrospective, and cross-sectional study. The study was carried out at the Kütahya Health Sciences University Hospital in Kütahya, Turkey. The diagnosis of COVID-19 was made using the polymerase chain reaction test (Rotor-Gene Q, Qiagen, Hilden, Germany). Patients' demographic characteristics, comorbidities, medical prescriptions, and laboratory test results were recorded. The time window for therapy administration was checked to assume the concomitant administration. Polypharmacy has defined as the use of at least five drugs daily 17.

### Drug-drug Interactions

The presence of pDDIs was identified by using the Lexi-Interact® database (Lexicomp Inc., Hudson, Ohio, USA) and categorized according to severity as A, B, C, D, and X risk categories. In the A risk category, data have not demonstrated either pharmacodynamic or pharmacokinetic interactions between the specified agents. In the B risk category, data demonstrate that the specified agents may interact with each other, but there is little to no evidence of clinical concern resulting from their concomitant use. In the C risk category, data demonstrate that the specified agents may interact with each other in a clinically significant manner. The benefits of concomitant use of these two medications usually outweigh the risks. An appropriate monitoring plan should be implemented to identify potential negative effects. Dosage adjustments of one or both agents may be needed in a minority of patients. In the D risk category, data demonstrate that the two medications may interact with each other in a clinically significant manner. A patient-specific assessment must be conducted to determine whether the benefits of concomitant therapy outweigh the risks. Specific actions must be taken to realize the benefits and/or minimize the toxicity resulting from the concomitant use of the agents. These actions may include aggressive monitoring, empiric dosage changes, choosing alternative agents. In the X risk category, data demonstrate that the specified agents may interact with each other in a clinically significant manner. The risks associated with the concomitant use of these agents usually outweigh the benefits. These agents are generally considered contraindicated. A and B risk category pDDIs are clinically insignificant and need no actions. However, C, D, and X risk category pDDIs are considered clinically important and need further analysis. It is recommended to monitor therapy for pDDIs in the C risk category and to modify the regimen for pDDIs in the D risk category. For pDDIs in the X risk category, the combination should be avoided.

This retrospective study involving human participants was in accordance with the ethical standards of the institutional and national research committee and with the 1964 Helsinki Declaration and its later amendments or comparable ethical standards. The Clinical Research Ethics Committee of the institution approved the study protocol (Date 26. June.2020/No. 2020/10-14).

### Statistical analysis

Statistical analyses were performed by using SPSS for Windows version 16.0 (SPSS, Chicago, IL, USA). Kolmogorov-Smirnov and Shapiro-Wilk tests are used for evaluating the normality of distributions. The results with normal distribution were expressed as mean ± standard deviation, while data with non-normal distribution were expressed as median with interquartile range (25th 75th percentiles) for continuous variables. Categorical variables were presented as a number with percentages. Correlation analysis was performed using Spearman's correlation analysis. P<0.05 was considered statistically significant.

## Results

One hundred and twenty-six patients with COVID-19 infection were included in the study. Demographics, clinical characteristics, and laboratory parameters of the patients are presented in Table 1. The age of the patients ranged from 26 to 87 years and the mean age was 58±13 years. Sixty-two patients were female (49.2%); 37 patients (29.4%) were 65 years or older. Forty-nine of the patients had hypertension (38.9%), 34 of the patients had diabetes mellitus (27%), 15 of the patients had hyperlipidemia (11.9%), 8 of the patients had coronary artery disease (6.3%), and 8 of the patients (6.3%) had chronic obstructive pulmonary disease. The clinical presentation of COVID-19 was upper respiratory tract infection in 12 (9.5%) patients, mild-moderate pneumonia in 99 (78.6%) patients, and severe pneumonia in 15 (11.9%) patients. The median length of hospital stay was 10 (6–13) days. Eight of the patients required intensive care treatment and the median length of the intensive care unit stay was 11 (7–13) days. While 122 of the patients (96.8%) were discharged, 4 patients (3.2%) died of respiratory failure due to COVID-19 pneumonia. All of the deceased patients were over 65 years old.

**Table 1:** Demographics, clinical characteristics, and laboratory parameters of the patients

	n = 126
Age, years	58±13
Female, n	62 (49.2%)
Hypertension, n	49 (38.9%)
Diabetes mellitus, n	34 (27%)
Hyperlipidemia, n	15 (11.9%)
Coronary artery disease	8 (6.3%)
Chronic obstructive pulmonary disease	8 (6.3%)
Smoking, n	9 (7.1%)
Length of hospital stay, days	10 (6–13)
Patients admitted to intensive care unit, n	8 (6.3%)
Deceased patients, n	4 (3.2%)
Number of medications, n	8 (6–11)
Polypharmacy, n	118 (93.6%)
Urea, mg/dL	32 (25–40)
Creatinine, mg/dL	0.95 (0.81–1.09)
Glomerular filtration rate, mL/min	81 (70–93)
Aspartate transaminase, U/L	24 (19–35)
Alanine transaminase, U/L	21 (13–31)
C-reactive protein, mg/L	11.6 (4.4–38.3)
D-dimer, ng/mL	467 (302–699)

Normally distributed data are presented as mean ± standard deviation and non-normally distributed data are presented as median with interquartile ranges (25^th^-75^th^ percentiles). Categorical variables were presented as the number of patients with percentage.

In the evaluation in the terms of polypharmacy, it was found that patients used 2 to 18 drugs daily. The median number of drugs used by the patients was 8 (6–11). Two patients (1.6%) were taking 2 drugs, 6 patients (4.8%) were taking 3–4 drugs, and 118 patients (93.6%) were taking 5 or more drugs daily.

Drugs used in the COVID-19 treatment and the mean numbers of clinically important pDDIs per drug are shown in Table 2. The most commonly used drug for anti-viral therapy was HCQ (n=123, 97.6%), following the recommendations of the COVID-19 treatment guidelines valid at the time the data belongs. HCQ was followed by oseltamivir (n=80, 63.5%) and favipiravir (n=44, 34.9%). One hundred and fifteen patients (91.3%) were using enoxaparin. A total of 605 pDDIs were identified. Of these pDDIs, 23 (3.8%) were A risk category interaction, 186 (30.7%) were B risk category interaction, 339 (56%) were C risk category interaction, 54 (8.9%) were D risk category interaction, and 3 (0.5%) were X risk category interaction. The prevalence of pDDIs in patients for A risk category was 18.3% (23 patients), for B risk category was 69.8% (88 patients), for C risk category was 54% (68 patients), for D risk category was 27.8% (35 patients), and for X risk category was 2.4% (3 patients). Two hundred and nine (34.5%) of the pDDIs were in the A and B risk categories and clinically insignificant. Three hundred and ninety-six (65.5%) of the pDDIs were clinically important pDDIs (categories C, D, and X), and 69 patients (54.8%) had at least one clinically important pDDIs. There was a significant positive correlation between the number of drugs prescribed to patients and the number of clinically important pDDIs (r=0.80, p<0.001). The mean number of pDDIs per patient was 0.2 for A risk category pDDIs, 1.5 for B risk category pDDIs, 2.7 for C risk category pDDIs, 0.4 for D risk category pDDIs, 0.02 for X risk category pDDIs, 3.1 for clinically important pDDIs, and 4.8 for all of the pDDIs. The most commonly observed pDDIs in the B risk category was between HCQ and azithromycin (70 patients or 57.9%). HCQ was also the most interacting drug in the C risk category and 101 pDDIs (29.8%) were observed. HCQ had 19 pDDIs with metformin, 16 pDDIs with beta-blockers, 13 pDDIs with acetylsalicylic acid, 10 pDDIs with insulin, 8 pDDIs with moxifloxacin, 7 pDDIs with gliclazide, 6 pDDIs with escitalopram, 5 pDDIs with linagliptin, 4 pDDIs with chlorpromazine, 3 pDDIs with dapagliflozin and pioglitazone, and one with vildagliptin, acarbose, repaglinide, rasagiline, haloperidol, levofloxacin, and empagliflozin in the C risk category. The most interacting drug in the D risk category was enoxaparin, and 25 pD-DIs (48.1%) were observed with enoxaparin. Enoxaparin had 12 pDDIs with acetylsalicylic acid, 6 pDDIs wth escitalopram, 3 pDDIs with diclofenac, 2 pDDIs with clopidogrel, and one pDDIs with duloxetine and ticagrelor. Ipratropium bromide had 2 X risk category pDDIs with tiotropium bromide and one X risk category pDDIs with pheniramine.

**Table 2:** Drugs used in the COVID-19 treatment and the mean numbers of clinically important potential drug-drug interactions (C, D, and X categories) per drug

Drug	Number of patients (n=126)	Mean numbers of clinically important pDDIs per drug
Hydroxychloroquine	123 (97.6%)	0.9
Enoxaparin	115 (91.2%)	0.4
Vitamin C	99 (78.6%)	0
N-acetyl cysteine	87 (69%)	0
Oseltamivir	80 (63.5%)	<0.1
Azithromycin	73 (57.9%)	0.4
Favipiravir	44 (34.9%)	<0.1
Piperacillin/tazobactam	23 (18.3%)	0
Meropenem	9 (7.1%)	0
Lopinavir/ritonavir	1 (0.8%)	0

pDDIs: potential drug-drug interactions.

Clinically important interacting drugs and the number of interactions are shown in Table 3. The drug classes with the most interactions were anti-diabetic drugs (n=200), anti-infective drugs (n=196), and cardiovascular system drugs (n=139), respectively. Clinically important pDDIs were most observed with HCQ (n=110), metformin (n=54), gliclazide (n=48), enoxaparin (n=47), insulin (n=45), hydrochlorothiazide (n=40), and acetylsalicylic acid (n=40). Two clinically significant pDDIs were observed with oseltamivir, the second most commonly used anti-viral drug in the study group, and only one with favipiravir, the third most commonly used anti-viral drug in the study group. Among the drugs used for COVID-19 therapy, the drugs with the highest mean number of clinically important pDDIs per drug were HCQ (0.9), enoxaparin (0.4), and azithromycin (0.4). Possible effects of certain pDDIs and the numbers of patients exposed to certain pDDIs are shown in Table 4.

**Table 3:** C, D, or X risk group interacting drugs and number of interactions

Drug classes	n	Drugs and number of interactions
Anti-diabetic drugs	200	Metformin (n=54), Gliclazide (n=48), Insulin (n=45), Dapagliflozin (n=13), Linagliptin (n=11), Pioglitazone (n=7), Vildagliptin (n=7), Acarbose (n=4), Repaglinide (n=4), Empagliflozin (n=4), Sitagliptin (n=3)
Anti-infective drugs	196	Hydroxychloroquine (n=110), Azithromycin (n=31), Moxifloxacin (n=30), Levofloxacin (n=11), Linezolide (n=5), Fluconazole (n=4), Oseltamivir (n=2), Favipiravir (n=1), Tigecycline (n=1), Cefpodoxime (n=1)
Cardiovascular system drugs	139	Hydrochlorothiazide (n=40), Ramipril (n=13), Perindopril (n=13), Furosemide (n=12), Metoprolol succinate (n=9), Candesartan (n=6), Amlodipine (n=5), Indapamide (n=5), Nebivolol (n=4), Spironolactone (n=4), Doxazosin (n=4), Trandolapril (n=3), Pentoxifylline (n=3), Olmesartan (n=3), Losartan (n=3), Verapamil (n=3), Diltiazem (n=3), Telmisartan (n=1), Propranolol (n=1), Bisoprolol (n=1), Carvedilol (n=1), Captopril (n=1), Lercanidipine (n=1)
Anti-thrombotic drugs	71	Enoxaparin (n=47), Clopidogrel (n=12), Warfarin (n=6), Ticagrelor (n=4), Apixaban (n=2)
Central nervous system drugs	69	Escitalopram (n=28), Chlorpromazine (n=8), Pramipexole (n=7), Rasagiline (n=7), Quetiapine (n=6), Levodopa/benserazide (n=6), Haloperidol (n=3), Duloxetine (n=2), Pyridostigmine (n=1), Trazodone (n=1)
Non-steroidal anti- inflammatory drugs	57	Acetylsalicylic acid (n=40), Diclofenac (n=11), Dexketoprofen (n=6)
Respiratory system drugs	23	Salbutamol (n=9), Formoterol (n=5), Ipratropium bromide (n=3), Tiotropium bromide (n=2), Theophylline (n=2), Salmeterol (n=2)
Vitamins and minerals	15	Vitamin D (n=9), Calcium carbonate (n=5), Magnesium oxide (n=1)
Gastrointestinal system drugs	5	Domperidone (n=2), Pantoprazole (n=1), Ranitidine (n=1), Metoclopramide (n=1)
Others	17	Alpha-lipoic acid (n=4), Atorvastatin (n=4), Tacrolimus (n=2), Colchicine (n=2), Mycophenolate mofetil (n=2), Pheniramine (n=1), Prednisolone (n=1), Methimazole (n=1)
Total	792	

**Table 4:** Possible effects of certain drug-drug interactions in the study population

Drug-drug interactions	Risk Category	Possible effects	Number of patients
Hydroxychloroquine-Azithromycin	B	QT prolongation	70
Hydroxychloroquine-Beta blockers	C	QT prolongation	16
Hydroxychloroquine-Moxifloxacin	C	QT prolongation	8
Hydroxychloroquine-Escitalopram	C	QT prolongation/ Hypoglycemia	6
Hydroxychloroquine-Metformin	C	Hypoglycemia	19
Hydroxychloroquine-Acetylsalicylic acid	C	Hypoglycemia	13
Hydroxychloroquine-Insulin	C	Hypoglycemia	10
Hydroxychloroquine-Gliclazide	C	Hypoglycemia	7
Enoxaparin-Acetylsalicylic acid	D	Hemorrhage	12
Enoxaparin-Escitalopram	D	Hemorrhage	6
Enoxaparin-Diclofenac	D	Hemorrhage	3
Ipratropium bromide-Tiotropium bromide	X	Anticholinergic effect	2
Ipratropium bromide-Pheniramine	X	Anticholinergic effect	1

## Discussion

In this study, we evaluated the pDDIs in inpatients with a diagnosis of COVID-19. The vast majority of pDDIs observed in our study were clinically important pDDIs. Three contraindicated interactions (X risk category) that were very rare compared to other pDDIs categories were the concomitant use of ipratropium bromide with tiotropium bromide and pheniramine. These combinations should be avoided due to the increased risk of the anticholinergic effect. About three-quarters of the patients had at least one clinically important pDDI.

COVID-19 is a disease that is evaluated in the serious disease category and requires the cooperation of various disciplines of medicine in its treatment. Treatment of COVID-19 consists of multiple drug regimens and this makes a predisposition for pDDIs. Also, medications prescribed for the management of comorbidities in COVID-19 patients contribute to pDDIs. pDDIs occurring in the course of anti-COVID-19 treatment could lead to adverse drug reactions, increasing the risk of hospitalization, prolonged time to recovery, or death in extreme cases. In an analysis of the first wave of COVID-19 in Spain, the vast majority of the real drug-drug interactions were associated with the most commonly used drugs, lopinavir/ritonavir and HCQ 11. Although HCQ was the most widely used drug in this analysis, the greatest number of pDDIs were associated with lopinavir/ritonavir. In our analysis, lopinavir/ritonavir was used in only one patient and the greatest number of pDDIs were observed with HCQ which was the most widely used anti-viral drug in this analysis. According to the results of a meta-analysis by Awortwe et al., pDDIs on the pharmacokinetic level were identified between the atazanavir and lopinavir/ritonavir and some drugs, used in the treatment of cardiovascular diseases such as antiarrhythmics and anti-coagulants possibly affecting the clinical outcome because of QT prolongation or hemorrhage 14.

HCQ, enoxaparin, and azithromycin were found to be the most interacting drugs in our analysis. The most commonly used antibiotic was azithromycin, which belongs to the macrolide family, and acts by binding to the 50S ribosomal subunit and inhibiting protein synthesis. Azithromycin is also used for the prevention of secondary bacterial infections and its immunomodulating action. Its immunomodulating effect comes from the ability to increase the secretion of interferon beta and interferon lambda, and by decreasing moderately tumor necrosis factor-alpha (TNF-α) levels 18. It is a weak CYP3A4 inhibitor with little or no expected clinically significant interactions.

The anti-viral mechanism of HCQ is not commonly understood. It is thought to inhibit the replication of enveloped viruses by blocking the pH-dependent entry of viruses into host cells or inhibiting glycosylation of envelope proteins. It has also anti-inflammatory and immunomodulating activity by regulating TNF-α, interferon, and other cytokine production 19. Fifty-seven-point nine percent of patients were using HCQ concomitantly with azithromycin, and 42.1% were using HCQ alone. Azithromycin and HCQ both have the potential to prolong the QT interval 20. QT prolongation and cardiotoxicity closely correlate with HCQ dose and duration 21. In particular, concomitant use of azithromycin and HCQ may lead to prolongation of the QT interval, which may predispose to ventricular arrhythmias, and treatment discontinuation 22. Simultaneous beta-blocker therapy with HCQ has an increased risk of critical QT prolongation 23. Drug-induced bradycardia is the common mechanism for QT prolongation following beta-blocker use 24. Concomitant use of chloroquine, HCQ, azithromycin, and lopinavir/ritonavir with antipsychotics is also associated with the risk of QT prolongation 25. In our analysis, 17 clinically important pDDIs were observed with antipsychotic drugs. Escitalopram, a selective serotonin reuptake inhibitor, was the most commonly used drug among the drugs that act on the central nervous system in our analysis. HCQ-Escitalopram interaction may lead to prolongation of the QT interval. The use of quick and simple algorithms that might help clinicians in the management of arrhythmic risk before and during treatment with the specific drugs used against SARS-CoV2 may be considered 26.

HCQ has favorable effects on glucose metabolism. HCQ improves both beta-cell function, adiponectin levels, and insulin sensitivity in non-diabetic individuals 27. Inflammation normally affects insulin and glucose metabolism via cytokines such as TNF-α and Interleukin 6, and predispose to hyperglycemia. A study has shown that when HCQ was given to one group for anti-inflammatory treatment in patients with rheumatoid arthritis and diabetes mellitus, and methotrexate was given to another group, the decrease in hemoglobin A1c was greater in the group receiving HCQ. This study suggests that HCQ reduces blood glucose through non-inflammatory mechanisms 28. On the other hand, it has also been shown that HCQ improves glycemic control in treatment-resistant diabetic patients 29. In diabetic COVID-19 patients, hypoglycemia may be observed with the use of HCQ. In our analysis, clinically important pDDIs were most observed with anti-diabetic drugs. Patients taking HCQ concomitantly with metformin, acetylsalicylic acid, insulin, gliclazide, and escitalopram should be closely monitored for the risk of hypoglycemia. To prevent hypoglycemia, it is important to adjust the dose of anti-diabetic drugs when HCQ had been added to the therapy and blood glucose monitoring should be made closely in this group 28.

Among the anti-diabetic drugs, the drug with the highest number of clinically important pDDIs observed was metformin, and the majority of these pDDIs were with HCQ. Metformin is one of the most commonly prescribed drugs for diabetes mellitus. In severe COVID-19 patients, the risk of metabolic acidosis may increase due to metformin given in diabetes mellitus treatment. The study by Montastruc et al. was shown that metformin administration with HCQ increased toxicity compared to HCQ administration alone in searches using the World Health Organization pharmacovigilance database, Vigibase 30. If sodium-glucose cotransporter-2 inhibitors such as dapagliflozin and empagliflozin are used as an oral anti-diabetic treatment in diabetic patients with COVID-19, they may aggravate the disease due to volume reduction 31.

The most common drug that interacts among the D risk category was enoxaparin, and the drugs that enoxaparin interacted most with were acetylsalicylic acid, escitalopram, and diclofenac. It is recommended to give low molecular weight heparin to all COVID-19 patients, including also not critically ill patients 32. Although acetylsalicylic acid is not widely recommended in guidelines for the treatment of COVID-19, it has been confirmed to have multiple levels of anti-viral effects 33. It inhibits prostaglandin E2 in the macrophages, increases type 1 interferon production, and therefore inhibits virus replication 34. Acetylsalicylic acid is not indicated for the treatment of disseminated intravascular coagulation or other venous thromboembolic complications associated with severe COVID-19 and may increase the risk of bleeding in severely thrombocytopenic patients 35. One study recommends risk stratification based on troponin and D-dimer values in COVID-19 patients when adding prophylactic acetylsalicylic acid to anticoagulant therapy 36. If acetylsalicylic acid is to be added to enoxaparin in the treatment of COVID-19, patients should be closely monitored for increased bleeding risk. The same suggestion applies to escitalopram, diclofenac, clopidogrel, duloxetine, and ticagrelor as well.

In a study conducted on over 600,000 elderly Swedish patients, a strong association was found between the number of dispensed drugs and the probability of type C and D pDDIs 37. A similar strong association between the number of drugs prescribed to patients and the number of clinically important pDDIs was found in our analysis results. In our study group, almost all of the patients were using five or more drugs daily. We consider that the high number of drugs prescribed to patients was the main reason for the high number of pDDIs. The increased number of dispensed drugs due to comorbidities may increase the severity and mortality of COVID-19 in affected patients by causing pDDIs 14. To avoid pDDIs during the treatment of COVID-19, reducing the number of drugs prescribed, dose adjustment of drugs predisposed to pDDIs, or using alternative drugs for the management of related comorbidities can be considered.

## Conclusion

The prevalence of COVID-19 patients having pDDIs was found to be high and is closely related to polypharmacy. The most prevalent pDDIs were C risk category interactions that need monitoring of therapy. HCQ was the most commonly used and most interacting drug. Favipiravir and oseltamivir were safer than HCQ in terms of clinically important pDDIs. COVID-19 patients should be closely observed for QT prolongation, hypoglycemia, and hemorrhage due to pDDIs during COVID-19 therapy. In particular, anti-diabetic drugs, anti-infective drugs, and drugs acting on the cardiovascular system should be used carefully during the COVID-19 therapy in terms of clinically important pDDIs.

## References

[1] Wang D, Hu B, Hu C, Zhu F, Liu X, Zhang J (2020). Clinical Characteristics of 138 Hospitalized Patients With 2019 Novel Coronavirus-Infected Pneumonia in Wuhan, China. JAMA.

[2] Zhou P, Yang XL, Wang XG, Hu B, Zhang L, Zhang W (2020). A pneumonia outbreak associated with a new coronavirus of probable bat origin. Nature.

[3] Huang C, Wang Y, Li X, Ren L, Zhao J, Hu Y (2020). Clinical features of patients infected with 2019 novel coronavirus in Wuhan, China. Lancet.

[4] Zhou F, Yu T, Du R, Fan G, Liu Y, Liu Z (2020). Clinical course and risk factors for mortality of adult inpatients with COVID-19 in Wuhan, China: a retrospective cohort study. Lancet.

[5] McMichael TM, Currie DW, Clark S, Pogosjans S, Kay M, Schwartz NG (2020). Epidemiology of Covid-19 in a Long-Term Care Facility in King County, Washington. N Engl J Med.

[6] Onder G, Rezza G, Brusaferro S (2020). Case-Fatality Rate and Characteristics of Patients Dying in Relation to COVID-19 in Italy [published correction appears in JAMA. 2020 Apr 28;323(16):1619]. JAMA.

[7] Schrezenmeier E, Dörner T (2020). Mechanisms of action of hydroxychloroquine and chloroquine: implications for rheumatology. Nat Rev Rheumatol.

[8] Farkas J (2020). Covid-19. Internet Book of Critical Care (IBCC).

[9] Republic of Turkey Ministry of Health (2020). COVID-19 Treatment Guidelines.

[10] World Health Organization (2020). Report of the WHO-China Joint Mission on Coronavirus Disease 2019 (COVID-19).

[11] Cantudo-Cuenca MD, Gutiérrez-Pizarraya A, Pinilla-Fernández A, Contreras-Macías E, Fernández-Fuertes M, Lao-Domínguez FA (2021). Drug-drug interactions between treatment specific pharmacotherapy and concomitant medication in patients with COVID-19 in the first wave in Spain. Sci Rep.

[12] Cattaneo D, Pasina L, Maggioni AP, Giacomelli A, Oreni L, Covizzi A (2020). Drug-Drug Interactions and Prescription Appropriateness in Patients with COVID-19: A Retrospective Analysis from a Reference Hospital in Northern Italy. Drugs Aging.

[13] Rezaee H, Pourkarim F, Pourtaghi-Anvarian S, Entezari-Maleki T, Asvadi-Kermani T, Nouri-Vaskeh M (2021). Drug-drug interactions with candidate medications used for COVID-19 treatment: An overview. Pharmacol Res Perspect.

[14] Awortwe C, Cascorbi I (2020). Meta-analysis on outcome-worsening comorbidities of COVID-19 and related potential drug-drug interactions. Pharmacol Res.

[15] Dookeeram D, Bidaisee S, Paul JF, Nunes P, Robertson P, Maharaj VR (2017). Polypharmacy and potential drug-drug interactions in emergency department patients in the Caribbean. Int J Clin Pharm.

[16] Aşcı H, Sönmez Y, Saygın M, Cankara F, Yeşilot Ş, Yıldırım M (2016). Investigation the presence of potential drug-drug interactions in adult intensive care unit: retrospective study. Med J SDU.

[17] Viktil KK, Blix HS, Moger TA, Reikvam A (2007). Polypharmacy as commonly defined is an indicator of limited value in the assessment of drug-related problems. Br J Clin Pharmacol.

[18] Menzel M, Akbarshahi H, Bjermer L, Uller L (2016). Azithromycin induces anti-viral effects in cultured bronchial epithelial cells from COPD patients. Sci Rep.

[19] Boulware DR, Pullen MF, Bangdiwala AS, Pastick KA, Lofgren SM, Okafor EC (2020). A Randomized Trial of Hydroxychloroquine as Postexposure Prophylaxis for Covid-19. N Engl J Med.

[20] Jankelson L, Karam G, Becker ML, Chinitz LA, Tsai MC (2020). QT prolongation, torsades de pointes, and sudden death with short courses of chloroquine or hydroxychloroquine as used in COVID-19: A systematic review. Heart Rhythm.

[21] Monzani A, Genoni G, Scopinaro A, Pistis G, Kozel D, Secco GG (2020). QTc evaluation in COVID-19 patients treated with chloroquine/hydroxychloroquine. Eur J Clin Invest.

[22] Murat B, Akgun H, Akarsu M, Ozmen A, Murat S (2021). The impact of hydroxychloroquine and azithromycin on the corrected qt interval in patients with the novel Coronavirus disease 2019. Rev Assoc Med Bras (1992).

[23] Haghjoo M, Golipra R, Kheirkhah J, Golabchi A, Shahabi J, Oni-Heris S (2021). Effect of COVID-19 medications on corrected QT interval and induction of torsade de pointes: Results of a multicenter national survey. Int J Clin Pract.

[24] Tisdale JE, Jaynes HA, Kingery JR, Mourad NA, Trujillo TN, Overholser BR (2013). Development and validation of a risk score to predict QT interval prolongation in hospitalized patients. Circ Cardiovasc Qual Outcomes.

[25] Plasencia-García BO, Rodríguez-Menéndez G, Rico-Rangel MI, Rubio-García A, Torelló-Iserte J, Crespo-Facorro B (202). Drug-drug interactions between COVID-19 treatments and antipsychotics drugs: integrated evidence from 4 databases and a systematic review. Psychopharmacology (Berl).

[26] Sciaccaluga C, Cameli M, Menci D, Mandoli GE, Sisti N, Cameli P (2021). COVID-19 and the burning issue of drug interaction: never forget the ECG. Postgrad Med J.

[27] Wasko MC, McClure CK, Kelsey SF, Huber K, Orchard T, Toledo FG (2015). Antidiabetogenic effects of hydroxychloroquine on insulin sensitivity and beta cell function: a randomised trial. Diabetologia.

[28] Rekedal LR, Massarotti E, Garg R, Bhatia R, Gleeson T, Lu B (2010). Changes in glycosylated hemoglobin after initiation of hydroxychloroquine or methotrexate treatment in diabetes patients with rheumatic diseases. Arthritis Rheum.

[29] Gerstein HC, Thorpe KE, Taylor DW, Haynes RB (2002). The effectiveness of hydroxychloroquine in patients with type 2 diabetes mellitus who are refractory to sulfonylureas--a randomized trial. Diabetes Res Clin Pract.

[30] Montastruc JL, Toutain PL (2020). A New Drug-Drug Interaction Between Hydroxychloroquine and Metformin? A Signal Detection Study. Drug Saf.

[31] Carboni E, Carta AR, Carboni E (2020). Can pioglitazone be potentially useful therapeutically in treating patients with COVID-19?. Med Hypotheses.

[32] Thachil J, Tang N, Gando S, Falanga A, Cattaneo M, Levi M (2020). ISTH interim guidance on recognition and management of coagulopathy in COVID-19. J Thromb Haemost.

[33] Glatthaar-Saalmüller B, Mair KH, Saalmüller A (2017). Antiviral activity of aspirin against RNA viruses of the respiratory tract-an in vitro study. Influenza Other Respir Viruses.

[34] Nakatsugi S, Sugimoto N, Furukawa M (1996). Effects of non-steroidal anti-inflammatory drugs on prostaglandin E2 production by cyclooxygenase-2 from endogenous and exogenous arachidonic acid in rat peritoneal macrophages stimulated with lipopolysaccharide. Prostaglandins Leukot Essent Fatty Acids.

[35] Léonard-Lorant I, Delabranche X, Séverac F, Helms J, Pauzet C, Collange O (2020). Acute Pulmonary Embolism in Patients with COVID-19 at CT Angiography and Relationship to d-Dimer Levels. Radiology.

[36] Mohamed-Hussein AAR, Aly KME, Ibrahim MAA (2020). Should aspirin be used for prophylaxis of COVID-19-induced coagulopathy?. Med Hypotheses.

[37] Johnell K, Klarin I (2007). The relationship between number of drugs and potential drug-drug interactions in the elderly: a study of over 600,000 elderly patients from the Swedish Prescribed Drug Register. Drug Saf.

